# Supplementary material for: Causal relationships between sex hormone traits, lifestyle factors, and osteoporosis in men: A Mendelian randomization study
Source: PLoS One. 2022 Aug 4;17(8):e0271898. doi: 10.1371/journal.pone.0271898 (PMC9351993; doi:10.1371/journal.pone.0271898)
Supplement: S1 File — (DOCX) [file pone.0271898.s001.docx]

**Table S1. Results of False Discovery Rate correction.**

| Exposure | Original P value | Adjusted P value | Significant or not |
| --- | --- | --- | --- |
| Heel bone |  |  |  |
| SHBG | 0.000 | 0.000 | Yes |
| TT | 0.018 | 0.032 | Yes |
| FT | 0.000 | 0.000 | Yes |
| E2 | 0.000 | 0.000 | Yes |
| Smoking | 0.742 | 0.742 | No |
| Drinking | 0.738 | 0.742 | No |
| BMI | 0.026 | 0.036 | Yes |
| Lumbar spine bone | |  |  |
| SHBG | 0.832 | 0.876 | No |
| TT | 0.876 | 0.876 | No |
| FT | 0.000 | 0.004 | Yes |
| E2 | 0.021 | 0.074 | No |
| Smoking | 0.842 | 0.876 | No |
| Drinking | 0.461 | 0.807 | No |
| BMI | 0.333 | 0.777 | No |
| Femoral neck bone | |  |  |
| SHBG | 0.059 | 0.138 | No |
| TT | 0.928 | 0.942 | No |
| FT | 0.053 | 0.138 | No |
| E2 | 0.942 | 0.942 | No |
| Smoking | 0.541 | 0.757 | No |
| Drinking | 0.018 | 0.126 | No |
| BMI | 0.419 | 0.733 | No |
| Forearm bone |  |  |  |
| SHBG | 0.000 | 0.003 | Yes |
| TT | 0.000 | 0.003 | Yes |
| FT | 0.823 | 0.936 | No |
| E2 | 0.803 | 0.936 | No |
| Smoking | 0.884 | 0.936 | No |
| Drinking | 0.936 | 0.936 | No |
| BMI | 0.211 | 0.491 | No |

SHBG sex hormone binding globulin, TT total testosterone, FT free testosterone, E2 estradiol, BMI the body mass index.

**Table S2. Mendelian randomization results of IVW, weighted median, MR-Egger and MR-PRESSO methods.**

|  |  | IVW | | Weighted median | | | MR-Egger | | | |  | |  | | MR-PRESSO | | | | | | | |
| --- | --- | --- | --- | --- | --- | --- | --- | --- | --- | --- | --- | --- | --- | --- | --- | --- | --- | --- | --- | --- | --- | --- |
|  | NSNP | OR (95% CI) | P | OR (95% CI) | P | | OR (95% CI) | | P | | P_heterogeneity_ | | P_pleiotropy_ | | OR (95% CI) | | | P | | | |  |
| Heel bone | | | | | |  | |  | |  |  |  | |  | |  | | | |  |  |  |
| SHBG | 162 | 0.87(0.83,0.91) | <0.001 | \| 0.87(0.83,0.91) \| \| --- \| | <0.001 | | \| 0.88(0.81,0.96) \| \| --- \| | | <0.001 | | <0.001 | | 0.613 | | \| 0.84(0.80,0.88) \| \| --- \| | | | <0.001 | | | |  |
| TT | 91 | 0.98(0.95,1.01) | 0.197 | \| 0.96(0.93,0.99) \| \| --- \| | 0.018 | | \| 0.92(0.86,0.99) \| \| --- \| | | 0.026 | | <0.001 | | 0.061 | | \| 0.98(0.95,1.00) \| \| --- \| | | | 0.158 | | | |  |
| FT | 56 | 1.16(1.13,1.19) | <0.001 | \| 1.13(1.10,1.17) \| \| --- \| | <0.001 | | \| 1.12(1.06,1.17) \| \| --- \| | | <0.001 | | <0.001 | | 0.087 | | \| 1.17(1.14,1.21) \| \| --- \| | | | <0.001 | | | |  |
| E2 | 5 | 2.51(1.58,3.98) | <0.001 | \| 2.07(1.33,3.23) \| \| --- \| | 0.001 | | \| 4.22(1.50,11.85) \| \| --- \| | | 0.072 | | 0.058 | | 0.352 | | \| 2.51(1.58,3.98) \| \| --- \| | | | 0.017 | | | |  |
| Smoking | 258 | 0.99(0.97,1.01) | 0.560 | \| 1.00(0.97,1.02) \| \| --- \| | 0.742 | | \| 1.01(0.93,1.10) \| \| --- \| | | 0.720 | | <0.001 | | 0.607 | | \| 0.99(0.97,1.01) \| \| --- \| | | | 0.449 | | | |  |
| Drinking | 68 | 1.01(0.95,1.07) | 0.878 | \| 1.01(0.93,1.10) \| \| --- \| | 0.738 | | \| 0.93(0.77,1.14) \| \| --- \| | | 0.504 | | <0.001 | | 0.443 | | \| 0.99(0.93,1.06) \| \| --- \| | | | 0.858 | | | |  |
| BMI | 21 | 1.10(1.05,1.15) | <0.001 | \| 1.06(1.01,1.12) \| \| --- \| | 0.026 | | \| 1.17(0.96,1.42) \| \| --- \| | | 0.133 | | <0.001 | | 0.543 | | \| 1.10(1.05,1.15) \| \| --- \| | | | <0.001 | | | |  |
| Lumbar spine bone | | | | |  | |  | |  | |  | |  | |  | |  | |  | | | |
| SHBG | 182 | 0.87(0.76,0.99) | 0.039 | \| 0.98(0.78,1.22) \| \| --- \| | 0.832 | | \| 0.78(0.62,0.99) \| \| --- \| | | 0.044 | | <0.001 | | 0.30 | | \| 0.89(0.78,1.01) \| \| --- \| | | | 0.075 | | | |  |
| TT | 112 | 0.97(0.89,1.06) | 0.501 | \| 1.01(0.90,1.13) \| \| --- \| | 0.876 | | \| 0.89(0.70,1.12) \| \| --- \| | | 0.325 | | <0.001 | | 0.425 | | \| 0.97(0.89,1.05) \| \| --- \| | | | 0.468 | | | |  |
| FT | 64 | 1.18(1.07,1.29) | <0.001 | \| 1.19(1.04,1.35) \| \| --- \| | 0.009 | | \| 1.31(1.03,1.65) \| \| --- \| | | 0.028 | | 0.060 | | 0.34 | | \| 1.16(1.06,1.27) \| \| --- \| | | | 0.002 | | | |  |
| E2 | 9 | 2.13(0.77,5.89) | 0.146 | \| 3.53(1.21,10.32)) \| \| --- \| | 0.021 | | \| 6.33(0.45,88.58) \| \| --- \| | | 0.213 | | 0.051 | | 0.409 | | \| 2.13(0.77,5.89) \| \| --- \| | | | 0.184 | | | |  |
| Smoking | 265 | 1.00(0.94,1.08) | 0.917 | \| 1.01(0.92,1.11) \| \| --- \| | 0.842 | | \| 0.89(0.66,1.19) \| \| --- \| | | 0.420 | | 0.001 | | 0.393 | | \| 1.00(0.94,1.07) \| \| --- \| | | | 0.948 | | | |  |
| Drinking | 66 | 0.91(0.72,1.16) | 0.461 | \| 0.79(0.57,1.11) \| \| --- \| | 0.178 | | \| 0.85(0.42,1.74) \| \| --- \| | | 0.659 | | 0.072 | | 0.838 | | \| 0.91(0.71,1.15) \| \| --- \| | | | 0.428 | | | |  |
| BMI | 14 | 1.09(0.92,1.30) | 0.333 | \| 1.23(0.97,1.57) \| \| --- \| | 0.092 | | \| 1.00(0.48,2.08) \| \| --- \| | | 0.994 | | 0.382 | | 0.819 | | \| 1.08(0.92,1.27) \| \| --- \| | | | 0.384 | | | |  |
| Femoral neck bone | | | |  |  | |  | |  | |  | |  | |  | |  | |  | | | |
| SHBG | 181 | 0.87(0.77,0.98) | 0.018 | \| 0.82(0.67,1.01) \| \| --- \| | 0.059 | | \| 0.88(0.71,1.08) \| \| --- \| | | 0.228 | | <0.001 | | 0.869 | | \| 0.88(0.78,0.99) \| \| --- \| | | | 0.041 | | | |  |
| TT | 114 | 1.00(0.94,1.06) | 0.928 | \| 1.00(0.91,1.09) \| \| --- \| | 0.932 | | \| 1.04(0.87,1.24) \| \| --- \| | | 0.686 | | 0.089 | | 0.639 | | \| 1.00(0.94,1.07) \| \| --- \| | | | 0.945 | | | |  |
| FT | 63 | 1.08(1.00,1.17) | 0.053 | \| 1.10(0.98,1.22) \| \| --- \| | 0.101 | | \| 1.27(1.06,1.54) \| \| --- \| | | 0.015 | | 0.260 | | 0.065 | | \| 1.08(1.00,1.16) \| \| --- \| | | | 0.056 | | | |  |
| E2 | 8 | 0.97(0.44,2.15) | 0.942 | \| 0.73(0.25,2.11) \| \| --- \| | 0.558 | | \| 0.90(0.10,8.26) \| \| --- \| | | 0.928 | | 0.319 | | 0.943 | | \| 0.97(0.44,2.15) \| \| --- \| | | | 0.944 | | | |  |
| Smoking | 264 | 0.98(0.93,1.04) | 0.541 | \| 0.95(0.87,1.03) \| \| --- \| | 0.195 | | \| 0.90(0.71,1.14) \| \| --- \| | | 0.396 | | 0.081 | | 0.468 | | \| 0.99(0.94,1.04) \| \| --- \| | | | 0.711 | | | |  |
| Drinking | 67 | 0.88(0.71,1.10) | 0.277 | \| 0.70(0.52,0.94) \| \| --- \| | 0.018 | | \| 0.76(0.40,1.46) \| \| --- \| | | 0.410 | | 0.011 | | 0.625 | | \| 0.86(0.69,1.08) \| \| --- \| | | | 0.193 | | | |  |
| BMI | 14 | 1.07(0.91,1.27) | 0.419 | \| 1.08(0.88,1.34) \| \| --- \| | 0.454 | | \| 1.33(0.67,2.65) \| \| --- \| | | 0.435 | | 0.210 | | 0.540 | | \| 1.06(0.91,1.24) \| \| --- \| | | | 0.480 | | | |  |
| Forearm bone | | | |  |  | |  | |  | |  | |  | |  | |  | |  | | | |
| SHBG | 201 | 0.75(0.64,0.88) | <0.001 | \| 0.70(0.53,0.92) \| \| --- \| | 0.009 | | \| 0.71(0.56,0.89) \| \| --- \| | | 0.004 | | 0.280 | | 0.458 | | \| 0.76(0.65,0.90) \| \| --- \| | | | 0.001 | | | |  |
| TT | 127 | 0.85(0.77,0.93) | <0.001 | \| 0.80(0.68,0.95) \| \| --- \| | 0.009 | | \| 0.86(0.72,1.02) \| \| --- \| | | 0.083 | | 0.274 | | 0.859 | | \| 0.85(0.77,0.93) \| \| --- \| | | | <0.001 | | | |  |
| FT | 68 | 0.94(0.80,1.12) | 0.498 | \| 1.03(0.81,1.31) \| \| --- \| | 0.823 | | \| 1.23(0.80,1.88) \| \| --- \| | | 0.350 | | 0.015 | | 0.191 | | \| 0.97(1.03,0.91) \| \| --- \| | | | 0.712 | | | |  |
| E2 | 9 | 0.80(0.14,4.60) | 0.803 | \| 1.14(0.19,6.76) \| \| --- \| | 0.888 | | \| 0.40(0.06,27.91) \| \| --- \| | | 0.683 | | 0.051 | | 0.730 | | \| 0.80(0.14,4.63) \| \| --- \| | | | 0.810 | | | |  |
| Smoking | 282 | 0.99(0.89,1.11) | 0.884 | \| 0.98(0.83,1.15) \| \| --- \| | 0.774 | | \| 1.28(0.80,2.03) \| \| --- \| | | 0.307 | | 0.223 | | 0.277 | | \| 0.99(0.89,1.10) \| \| --- \| | | | 0.905 | | | |  |
| Drinking | 70 | 0.99(0.68,1.42) | 0.936 | \| 0.70(0.40,1.22) \| \| --- \| | 0.205 | | \| 1.43(0.50,4.03) \| \| --- \| | | 0.507 | | 0.414 | | 0.459 | | \| 0.98(0.69,1.40) \| \| --- \| | | | 0.913 | | | |  |
| BMI | 19 | 1.21(0.90,1.63) | 0.211 | \| 1.18(0.83,1.68) \| \| --- \| | 0.361 | | \| 1.51(0.44,5.19) \| \| --- \| | | 0.523 | | 0.143 | | 0.722 | | \| 1.11(0.80,1.54) \| \| --- \| | | | 0.529 | | | |  |

NSNP number of single nucleotide polymorphism, OR odds ratio, 95%CI lower and upper limit of 95% confidence interval, P p-value of OR, P_heterogeneity_ p-value of Cochrane’s Q value in heterogeneity test, P_pleiotropy_ p-value of MR-Egger intercept, SHBG sex hormone binding globulin, TT total testosterone, FT free testosterone, E2 estradiol, BMI body mass index.
